# Advanced Composites Based on Sea Buckthorn Carotenoids for Mayonnaise Enrichment

**DOI:** 10.3390/polym14030548

**Published:** 2022-01-28

**Authors:** Diana Roman, Nina Nicoleta Condurache (Lazăr), Nicoleta Stănciuc, Doina Georgeta Andronoiu, Iuliana Aprodu, Elena Enachi, Vasilica Barbu, Gabriela Elena Bahrim, Silvius Stanciu, Gabriela Râpeanu

**Affiliations:** Department of Food Science, Food Engineering, Biotechnology and Aquaculture, Faculty of Food Science and Engineering, Dunărea de Jos University of Galati, 800008 Galati, Romania; diana.roman@ugal.ro (D.R.); nina.condurache@ugal.ro (N.N.C.); nicoleta.stanciuc@ugal.ro (N.S.); georgeta.andronoiu@ugal.ro (D.G.A.); iuliana.aprodu@ugal.ro (I.A.); elena.ionita@ugal.ro (E.E.); vasilica.barbu@ugal.ro (V.B.); gabriela.bahrim@ugal.ro (G.E.B.)

**Keywords:** sea buckthorn, carotenoids, microencapsulation, coacervation, mayonnaise, functional food

## Abstract

This study aimed at the extraction and encapsulation of the carotenoids from sea buckthorn fruits and obtaining value-added mayonnaise. First, the carotenoids from sea buckthorn fruits were extracted using ultrasound-assisted extraction. Then, they were microencapsulated through complex coacervation and freeze-drying techniques using different wall material combinations. Two powders were obtained and analyzed in terms of encapsulation efficiency, total carotenoid content, antioxidant activity, stability of phytochemicals and color, morphological structure, and in vitro digestibility. All results pointed out that the carotenoid molecules were successfully encapsulated within the mixture of alginate, agar, and chitosan, with a 61.17 ± 0.89% encapsulation efficiency. To probe the functionality, the powder was added into mayonnaise in 2.5% and 5% amounts. The obtained mayonnaise samples were characterized in terms of phytochemical and antioxidant activity properties with their storage stability and texture, color, and sensory characteristics. A significant increase of total carotenoid content and antioxidant activity compared to the control sample was observed. The addition of powder also led to improved texture by increasing the firmness and adhesion. In addition, the sensory evaluation indicated an improved color and overall acceptability of the value-added mayonnaise. Thus, sea buckthorn extracts may be considered as valuable ingredients for the development of added-value food products.

## 1. Introduction

The increasing occurrence of severe illnesses and their discovery in critical stages have led people to be more careful about food, sports, and health in general. Cancer, cardiovascular diseases, diabetes, and obesity are diseases that people suffer from the most. These conditions are mainly based on negligence in terms of lifestyle but are also due to heredity or other factors [1]. Therefore, in recent years, efforts have been made to raise public awareness of proper nutrition and the importance of exercise. Due to this, there has been an increase in the market demand for healthy, functional foods.

Banwo et al. [1] define functional food as a product full of compounds with biological and physiological activities that have the ability to interact with one or more components from living tissue and thus provide a wide range of potential health effects. These biologically active compounds usually refer to those produced by plants through primary and secondary metabolism. For some time now, most studies have focused on the second one. These products of secondary metabolism comprise compounds with essential roles in metabolism, photosynthesis, development, defense, and protection from photo-oxidative damage [2]. Examples of bioactive compounds include carotenoids, which are natural pigments, but they have a lack of stability and low bioavailability.

Sea buckthorn is a small tree belonging to the *Elaeagnaceae* family, and it is naturally distributed around the world. According to Ciesarová et al. [3], 150 sea buckthorn varieties have been identified, but among them, *Hippophae* *rhamnoides* is the most important and widely distributed. The berries are rich in carotenoids besides vitamin C, such as flavonoids and phytosterols, all of which are valuable for their nutritional and health-promoting properties. Carotenoids are important for their antioxidant activities, such as oxygen radical scavenging and singlet oxygen quenching [4]. Their consumption can trigger some mechanisms that can lead to beneficial effects on the body. For instance, Xiao et al. [5] explored the sea buckthorn flavonoid’s mechanism of action on hyperlipidemia. Likewise, Masoodi et al. [6] proved that sea buckthorn extracts could inhibit cell proliferation and decrease expression of a specific antigen in prostate cancer cells using in vitro tests. Many studies have addressed the subject of the multitudes of beneficial effects of sea buckthorn on the human body [7,8,9,10].

Despite all the advantages and benefits they bring, the direct incorporation of plant extracts in processed food is challenging. The usual process conditions or the interaction with other food constituents can lead to the degradation of carotenoids. The encapsulation technique is the best approach to increase their stability and bioavailability. The encapsulation process was defined by de Freitas Santos et al. [11] as any process that ensures a specific substance’s stability by its entrapment in another material, thus avoiding contact with any factor that can lead to degradation.

Sauces are consumed worldwide, and they are associated with various foods due to their positive influence on taste and color. The best-known sauce is mayonnaise. Mayonnaise represents a cold oil-in-water emulsion composed of mainly egg yolk and vegetable oil. From a nutritional point of view, it has high-energy value due to the fats used, but it also brings vitamins, proteins, and minerals [12]. Mayonnaise is rich in unsaturated fatty acids, which makes it sensitive to the oxidation process. This process could release toxic compounds such as free radicals that can affect the consumer’s health, sensory properties, and stability during storage. The oxidation of mayonnaise could be prevented by the addition of natural antioxidants from plant extracts [12].

The purpose of the present study was to extract and encapsulate the carotenoids from sea buckthorn fruits and obtain value-added mayonnaise. The use of sea buckthorn fruit extract in the composition of mayonnaise can contribute to the increased nutritional value of the product and therefore the quality of life. Hence, the objectives of this study were first to extract the carotenoids from sea buckthorn fruits, then to encapsulate them through coacervation and freeze-drying using different wall materials. In the end, the study aimed to obtain a value-added mayonnaise by the incorporation of the encapsulated sea buckthorn extract. The powders were characterized in terms of encapsulation efficiency, phytochemical content, antioxidant activity, phytochemical stability, color, morphological structure, and in vitro digestibility. The obtained mayonnaise samples were characterized in terms of phytochemical stability and antioxidant activity with their storage, texture, color, and sensory properties.

## 2. Materials and Methods

### 2.1. Chemicals

Hexane, ethanol, and methanol HPLC-grade sodium carbonate (Na_2_CO_3_), 2,2′-azino-bis (3-ethylbenzothiazoline-6-sulfonic acid) diammonium salt (ABTS), Trolox, whey protein isolate (WPI), carboxymethyl cellulose (CMC), alginate, agar, and chitosan were purchased from Sigma-Aldrich (Steinheim, Germany).

### 2.2. Extraction of Carotenoids from Sea Buckthorn Berries

Sea buckthorn was purchased from a local market in Galati, Romania, in March 2021. The sea buckthorn berries were washed, blotted with paper towels, frozen, and then lyophilized. The freeze-drying process took place at −42 °C with a pressure of 0.1 mBar for 72 h, using Alpha 1–4 LD Plus equipment from Christ (Osterode am Harz, Germany). Dried sea buckthorn fruits were stored in plastic bags with a lid at room temperature until extraction.

The carotenoids from sea buckthorn berries were extracted with ethanol and n-hexane (4:3, *v/v*), using the ultrasound-assisted extraction method described by Zu et al. [13], with slight modifications. Briefly, sea buckthorn freeze-dried berries were mixed with the solvent mixture (1:5, *w/v*) and treated with ultrasounds (Smart MRC LTD, Holon, Israel) for 20 min at 40 °C and a frequency of 40 kHz. Next, the extracts were centrifuged for 10 min at 5000 rpm and 4 °C. Further, the supernatant was collected and concentrated under reduced pressure at 40 °C using AVC 2–18 equipment from Christ (Osterode am Harz, Germany). Finally, the concentrated extracts were characterized.

#### 2.2.1. Total Carotenoid Content of Sea Buckthorn Extract

The extract was analyzed in terms of total carotenoids (TC) using spectrophotometric methods. The TC content was measured and calculated using the spectrophotometric method described by Mihalcea et al. [14]. Briefly, the extract was solubilized in the same extraction solvent mixture, and the absorbance was read at λ = 470 nm using a Libra S22 UV–VIS spectrophotometer (Biochrom, Cambridge, UK). The results were expressed as mg/g of dry weight (dw), using a β-carotene calibration curve. 

#### 2.2.2. In Vitro Antioxidant Activity of Sea Buckthorn Extract

The antioxidant activity of the extract was tested against ABTS free radical, and the results were expressed as µM Trolox equivalents (TE)/g dw using a calibration curve. The ABTS scavenging activity was measured according to Re et al. [15]. Thus, 1 mL of ABTS stock solution (7 mM) was mixed with 0.10 mL of extract solution and kept for 7 min at room temperature in the dark. The absorbance of the solution was measured at 734 nm against blank with a Libra S22 UV–VIS spectrophotometer (Biochrom, Cambridge, UK).

### 2.3. Microencapsulation of Total Carotenoids from Sea Buckthorn Berries Extract

The extract was encapsulated in two different matrices: one formed by 2% WPI and 2% CMC and one composed of 4% alginate, 1% agar, and 4% chitosan, in order to obtain two powders. These concentrations of biopolymers were chosen according to a preliminary study in which different concentrations were tested (data not shown) to find suitable ones. The encapsulation method used was the complex coacervation method described earlier by Mihalcea et al. [14], followed by freeze-drying. The extract was dissolved in sunflower seed oil (1:10, *w/v*) using ultrasounds for 1 h at 40 kHz and 40 °C (Smart MRC LTD, Holon, Israel). Withal, the wall materials were dissolved in distilled water using a magnetic stirrer at 700 rpm for 30 min at room temperature. Subsequently, the extract was combined with the matrix mixtures. The final mixture’s pH was adjusted to 4.00 with 1 N HCl solution to begin the coacervation process. Further, the mixtures were separated in a funnel and were collected, frozen, and lyophilized at −42 °C, under a pressure of 10 Pa for 72 h (Alpha 1–4 LD plus, Christ, Osterode am Harz, Germany). Finally, both powders were collected, packed in glass jars covered in aluminum foil, and stored in the refrigerator at 4 °C until further analysis.

#### 2.3.1. Powders Characterization and Encapsulation Efficiencies

The encapsulated powders were evaluated regarding the total carotenoid encapsulation efficiencies and initial total carotenoid content. The antioxidant activity was also measured.

The carotenoid’s encapsulation efficiency was determined based on Mihalcea et al. [14] method that quantifies total and surface carotenoids. The TC was assessed by dissolving 100 mg of encapsulated powder in 6 mL of 10% NaCl: methanol (1:1 ratio). The mixture was allowed to rest for 30 min, and then 30 mL of hexane was added. The mixture was treated with ultrasounds for 40 min at 50 °C and 40 kHz and afterward centrifuged at 6000 rpm for 10 min at 10 °C. The TC was quantified by reading the absorbance at λ = 470 nm using the same spectrophotometer mentioned above. 

The surface carotenoids (SC) were quantified by dissolving 100 mg of each powder in 30 mL of n-Hexane, followed by 2 min of vortex. Then, the samples were centrifuged at 6000 rpm for 10 min at 10 °C. The SC was quantified by reading the absorbance at λ = 470 nm using the same spectrophotometer mentioned above. 

The encapsulation efficiencies (EE) were calculated using the Equation:

EE (%) = (TC − SC)/TC × 100 (1)

TC = total carotenoids, mg/g dw; SC = surface carotenoids, mg/g dw.

#### 2.3.2. Confocal Laser Scanning Microscopy of Powders

The morphology and structure of freeze-dried powders were investigated by using the confocal laser scanning microscopy (CLSM) technique at a high resolution, as described by Condurache et al. [16]. In order to visualize the powders’ particles by CLSM, the samples were observed both in their native state and stained with Red Congo (40 μM). The distribution of the sea buckthorn bioactives into the matrices was analyzed using a Zeiss Axio Observer Z1 inverted microscope (40× apochromatic objective). The Zeiss confocal laser scanning system (LSM 710, Carl Zeiss, Oberkohen, Germany) used for the analysis the following lasers: a diode laser (405 nm), Ar-laser (458, 488, 514 nm), DPSS laser (561 nm), and HeNe-laser (633 nm). The resulting 3D images were analyzed and rendered using the ZEN 2012 SP1 software (black edition, Carl Zeiss, Oberkohen, Germany).

#### 2.3.3. CIELAB Color Characterization of Powders

The colors of the encapsulated particles were measured in terms of the CIE L*, a*, b* values, using a CR 410 Chroma Meter (Konica Minolta, Hino, Tokyo, Japan), as described by Idham et al. [17]. The analysis expresses color as L* for perceptual lightness or clarity (=0 black) or (=100 white) and a* and b* for the four unique colors of human vision: red, green, blue, and yellow. A* values express shades of green (<0) or red (>0) and the b* values shades of blue (<0) or yellow (>0).

#### 2.3.4. Carotenoid’s In Vitro Simulated Digestion

The microcapsules obtained were digested using a simulated gastrointestinal digestion model. Digestion media were prepared according to Oancea et al. [18]. Briefly, the powders were mixed with 0.1 M Tris HCl buffer solution (pH = 7.0) in a 1:1 ratio. Then, the mixtures were combined with simulated gastric juice (SGJ-20 mg pepsin in 0.1 M HCl, pH = 3.0) in a 1:1 ratio. Afterward, the mixtures were incubated at 37 °C, 150 rpm, for 2 h (Medline Scientific, Oxon, UK). Further, over the incubated samples in SGJ, simulated intestinal juice (SIJ-40 mg pancreatin in 0.9 M baking soda, pH = 7.0) was added in a 1:1 ratio. The mixtures were again allowed to incubate for 2 h at 37 °C and 150 rpm [19]. Every 30 min, 1 mL aliquots of each sample were taken and diluted with 2 mL of n-hexane, and the absorbance was read again at 450, 470, and 503 nm. Samples were prepared in triplicate (*n* = 3).

#### 2.3.5. Storage and Color Stability of Powders

The microparticles were kept at a controlled room temperature in the absence of light for 90 days. The variability of the relative humidity was set at the maximum possible. Phytochemical characteristics, as well as the color, were evaluated during the storage period with the methods previously described.

### 2.4. Mayonnaiese Formulation

Mayonnaise was obtained from the following ingredients: sunflower seed oil (80%), egg yolk powder (10%), salt (0.5%), powders from microencapsulated sea buckthorn fruits extract (2.5% and 5%), and water (until 100%). First, the egg yolk powder was dissolved in warm water (40–50 °C) along with the salt, in order to obtain an emulsion. Then, the sunflower oil was incorporated by continuous homogenization until the composition was uniform in color and texture. Afterward, the microencapsulated extract was added in different percentages. Thus, three mayonnaise variants were obtained: Control (without microencapsulated extract), Mayo 1, and Mayo 2 with 2.5% and 5% microencapsulated extract. After preparation, the control and enriched mayonnaises were stored at 4 °C for further analysis.

#### 2.4.1. Mayonnaise Characterization

The resulting mayonnaises sauce variants were analyzed in terms of physico-chemical and phytochemical content and color characteristics. Thus, the moisture, protein, lipid, ash, and total sugar content were analyzed according to AOAC methods [20]. Based on the protein, lipid, and sugar content, the energy values were calculated. The total carotenoids and antioxidant activity were assessed as described above. Moreover, the color parameters in terms of the CIE L*, a*, b* values were measured as described above.

#### 2.4.2. Texture Analysis of Value-Added Mayonnaise

The texture of value-added mayonnaise sauce was assessed using the texture profile analysis (TPA) method described by Horincar et al. [21]. Briefly, the mayonnaise samples were put in acrylic cylinders of 25.4 mm diameter. With a probe, double penetration into the sauces occurred until 10 mm depth at 1 mm/s speed, the trigger load being 0.067 N and the load cell 9.8 N. Determinations were performed in duplicate. The results were processed using TexturePro CT V1.5 software and firmness, adhesion, cohesiveness, and chewability were the calculated parameters.

Firmness is defined as the maximum resistance of the sample during the first penetration cycle with the test instrument. Adhesion is the energy required to remove the sample from the test instrument. Cohesion is a dimensionless quantity and is defined as the strength of the internal bonds that form and give the consistency of the tested product. Chewing refers to the energy required to chew food until it is swallowed [21].

#### 2.4.3. Sensorial Analysis of Value-Added Mayonnaise

The impact that a product has on consumers is one of the most significant characteristics. The sensory analysis must be present when we talk about a new product with added value. Therefore, the mayonnaise sauces were analyzed using a nine attributes scale based on a unit numbering. The followed characteristics are the color, aroma, taste, consistency, texture, smell, aftertaste, spreadability, and acceptability. Sensorial analysis was performed at 20 °C, under white light, and 45–47% relative air humidity by a panel of 20 tasters. After each sample, the participants rinsed their mouths with plain water. The 20 tasters were untrained consumers with ages ranging from 25 to 55 years old (80% women and 20% men). They received specific training on the sensory attributes relevant to the mayonnaise sauce formulation developed in our study using a range of commercial products. The sample preparation method and its safety for the body were explained to obtain written consent before participation. 

### 2.5. Statistical Analysis of Data

All analyses were performed in duplicate, and the results were statistically analyzed using Minitab 17 software. One-way ANOVA−Tukey test was used to assess the differences between the samples. All experimental data were expressed as the mean (*n* = 3) ± SD.

## 3. Results and Discussion

### 3.1. Sea Buckthorn Fruits Extract and Microcapsules Characterization

The results for the extract and powders characterization are presented in Table 1.

Table 1 shows that sea buckthorn fruit extract had a high carotenoid content of 32.33 ± 1.52 mg/g extract and antioxidant activity of 422.03 ± 1.33 µM TE/g extract. Our results comply with the ones obtained by Ursache et al. [4,22]. They reported a total carotenoid content of 38.34 ± 5.71 mg/g extract [22] and antioxidant activity of 449.85 ± 0.03 µM TE/g dw [4] after the conventional extraction of sea buckthorn fruit bioactives with ethanol and hexane.

In our study, the bioactive compounds from the extract were encapsulated by complex coacervation, followed by freeze-drying. The obtained powders showed average encapsulation efficiencies (Table 1). These findings are in good agreement with other studies. In this regard, Mihalcea et al. [23] reported encapsulation efficiency (EE) values between 41.34 ± 0.07% and 46.18 ± 0.13% after the encapsulation of sea buckthorn extract in WPI, acacia gum, and peptides obtained with transglutaminase. In another study, Mihalcea et al. [14] reported lower sea buckthorn bioactive EE of 36.23 ± 1.58% by also using WPI and acacia gum. Contrariwise, Drozinska et al. [24] reported higher sea buckthorn bioactive EE by using different combinations of β-glucan, Arabic gum, and maltodextrin, with values ranging from 61.34 ± 0.80% to 72.22 ± 2.85%.

From our results, it is observed that the combination of alginate, agar, and chitosan (P2) entrapped higher amounts of carotenoids from sea buckthorn extract, also presenting significantly higher EE values than the variant with WPI and CMC (*p* < 0.05). This difference between the powders is also reflected in the antioxidant activity (Table 1). Thus, values of 2.20 ± 0.13 mg/g dw and 2.89 ± 0.02 mg/g dw for total carotenoids were obtained, with similar values reported by Ursache et al. [4]. In terms of antioxidant activity, our powders presented values of 383.73 ± 2.14 µM TE/g dw and 454.04 ± 3.67 µM TE/g dw, respectively. Both Mihalcea et al. [14] and Ursache et al. [4] reported higher antioxidant activities such as 473.90 ± 5.01 µM Trolox/g dw and 548.00 ± 0.23 µM Trolox/g dw. Contrariwise, Souza et al. [25] reported lower antioxidant activity, with values between 4.29 ± 0.10 µM Trolox/g dw and 27.24 ± 11.28 µM Trolox/g dw for tomato extracts encapsulated in different ratios of maltodextrin, WPI, and modified starch.

### 3.2. Morphological Structure Analysis of Microcapsules by CLSM

**Figure 1:**
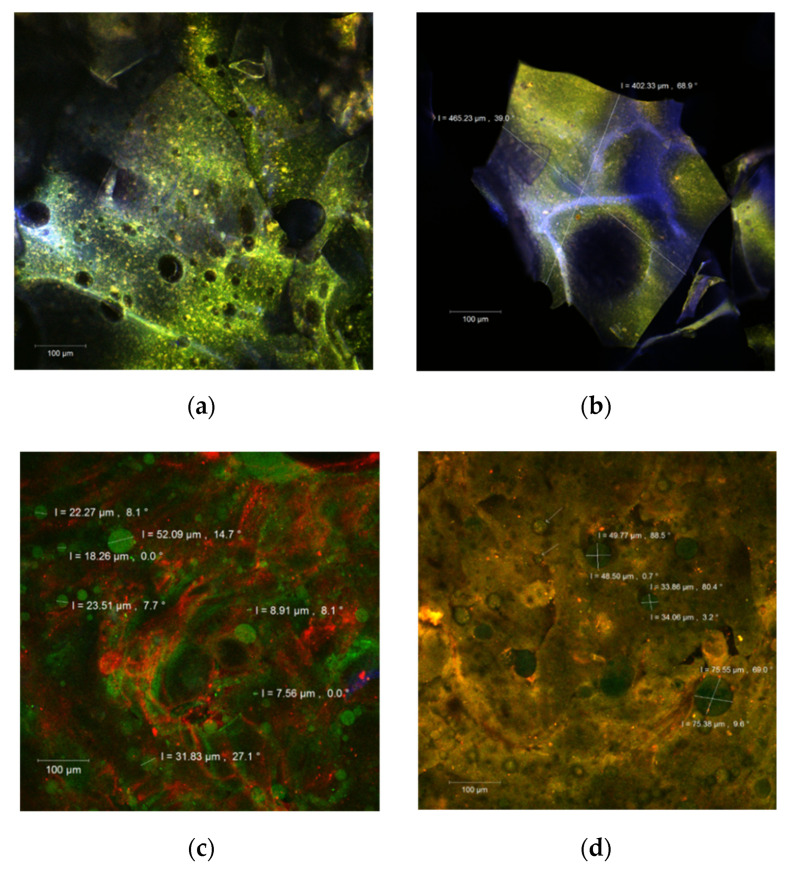
The confocal laser scanning microscopy (CLSM) images of the native, unstained (**a**,**b**), and fluorophore dyed powders (**c**,**d**).

The morphology of the microcapsules was evaluated by point-by-point laser scanning microscopy, and the resulting 3D images are presented in Figure 1. In the native form, fine biofilms of blue or green color were formed according to the WPI or polysaccharides ratio. The carotenoids from sea buckthorn extract were anchored within the encapsulation matrix as yellow to orange microspherosomes (1–2 µm) (Figure 1a,b). These observations are in good agreement with Ursache et al. [4] and Mihalcea et al. [14,23].

After staining with Red Congo fluorophore (Figure 1c,d), the carotenoids from the extract appear as green medium size spherosomes in both our powders. They seem well individualized and stabilized in the encapsulation matrices but with a tendency to group in clusters. Drozinska et al. [24] reported similar observations in a study in which they also used scanning electron microscopy to analyze the microcapsules of sea buckthorn extract.

### 3.3. Powders’ Color Analysis

Color data of the microparticles are shown in Table 2. Significant differences were observed between the powders for all colorimetric parameters (*p* < 0.05). These findings suggest that the encapsulating carrier system had a notable influence on the surface color of powders (*p* < 0.05). The L* and b* values suggest that both powders have a high lightness and yellowness, specific to carotenoid compounds. It can be stated that, in our case, the combination of polysaccharides used as wall material gave a significantly higher lightness than WPI and CMC (*p* < 0.05). Thus, P2 represents the lightest and yellowest sample between the two obtained but less red than P1. In addition, the significantly higher yellowness value of the P2 powder compared to P1 can be correlated with the higher carotenoid concentration (*p* < 0.05). Our results comply with Drozinska et al. [24], Ursache et al. [4], and Mihalcea et al. [14,23].

### 3.4. In Vitro Simulated Digestibility of Total Carotenoids from the Powders

**Figure 2:**
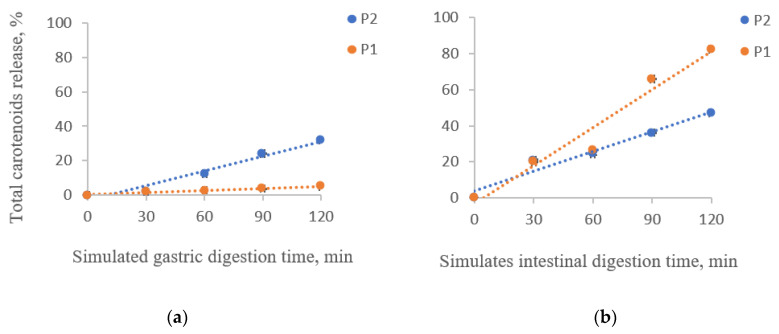
Total carotenoids release from the powders during the in vitro simulated gastric (**a**) and intestinal digestion (**b**). P1 = powder with 2% WPI and 2% CMC; P2 = powder with 4% alginate, 1% agar, and 4% chitosan.

The percentage of the released carotenoids in the digested sample solutions under simulated gastrointestinal digestion for two hours is shown in Figure 2.

A significant release of carotenoids from the matrix was observed in the gastric phase for P2, with a maximum of 32.07 ± 0.05% after 60 min of digestion (Figure 2a). P1 presented the highest stability in the simulated gastric phase, having a maximum release of carotenoids of 4.94 ± 0.07% after 120 min of digestion (Figure 2b). It is observed that although P2 showed the highest encapsulation efficiency, it has slightly lower stability in SGF than P1.

The carotenoids from both powders were released in the intestinal environment (Figure 2b). P1 presented a maximum release of 82.47 ± 0.22%, while P2 presented a 47.14 ± 0.44% release after 2 h of digestion. Hence, the carotenoid microencapsulation in a mixture of proteins and polysaccharides delayed the release in the gastric phase and enhanced it in the intestinal phase. Neagu et al. [26] also reported a low release in the gastric phase of the total carotenoids from sea buckthorn extracts encapsulated in WPI and casein. However, in the intestinal phase, they observed only a 42% release. 

### 3.5. Phytochemical and Color Stability of Powders

For evaluating the stability of microencapsulated carotenoids from sea buckthorn, the powders were stored in the dark at 4 °C for 90 days. Every 30 days, the total carotenoid content, antioxidant activity, and color parameters were measured. Table 3 presents the phytochemical characterization of the stored powders. 

Table 3 shows the total carotenoid content and antioxidant activity variations during 90 days of storage. A significant degradation of carotenoids occurred in both samples (*p* < 0.05). A 30% decrease in the TC content and 13% in the antioxidant activity of P1 took place, probably due to the weak links that formed between the protein and polysaccharide, which resulted in a possible oxidation process. A significant decrease in the TC content and antioxidant activity was also observed in P2 up to 13% and 11%, respectively (*p* < 0.05). From Table 3, it can also be noted that the polyglucide mixture in the encapsulation matrix from P2 provided better stability than the mixture between proteins and polysaccharides (P1). Thus, after 90 days of storage, P2 remains the powder with the highest TC and antioxidant activity. Our findings comply with other studies, who also reported carotenoids degradation after the storage of different microencapsulated plant extracts [27,28,29].

Color changes during storage were also measured, and the parameter modifications are presented in Table 4. 

Table 4 shows that the storage period and encapsulating agent significantly influenced the color change in both powders. A gradual degradation of yellowness was visually observed in both powders during 90 days of storage, highlighted by the significant decrease of b* parameter values (*p* < 0.05). However, the lightness and redness of both powders significantly increased during the storage time (*p* < 0.05). Thereby, in P1, the yellowness decreased by 13%, while in P2 by 7%. The yellowness degradation is correlated with carotenoid degradation. Instead, the lightness and redness increased by 20% and 18% in P1 and 9% and 38% in P2.

### 3.6. Value-Added Mayonnaise Characterization

Mayonnaise is probably one of the most well-known and consumed sauces worldwide. It is suitable for different types of food, improving their sensory characteristics. In our study, mayonnaise was obtained by adding into the classic recipe different percentages of encapsulated sea buckthorn extract. The chosen powder was P2 due to the higher content and stability of phytochemicals over time, having also the highest encapsulation efficiency. Thus, three variants with 0, 2.5, and 5% encapsulated microcapsules were obtained, coded as follows: Control, 0% powder; Mayo 1, 2.5% powder; Mayo 2, 5% powder. All samples were analyzed in terms of physico-chemical (Table 5) and phytochemical characteristics (Table 6).

The results presented in Table 5 indicate that the mayonnaise sauces with sea buckthorn powder added are characterized by a significantly lower protein content than the control one (*p* < 0.05). In contrast, the carbohydrates and lipids contents of the value-added sauces are similar to the control. In terms of energy value, there is a decrease of 1.53% at higher concentrations of powder. However, the energy values of all variants are very close.

The phytochemical content and antioxidant activities by the use of the ABTS method of value-added mayonnaise are presented in Table 6.

As expected, there is a significant increase in the concentration of carotenoids and antioxidant activity value as the concentration of added microcapsules increases (*p* < 0.05). Thus, the mayonnaise variants presented a TC content with values between 0.26 ± 0.07 mg/100 g dw and 1.85 ± 0.04 mg/100 g dw. As for the antioxidant activity, it has increased from 9.99 ± 0.60 µmol Trolox/g dw to 293.38 ± 2.77 µmol Trolox/g dw. The results presented in Table 6 confirm the added value of mayonnaise sauces with sea buckthorn powder by increasing the content of total carotenoids and antioxidant activity, respectively. These findings are in good agreement with that of Ursache et al. [4], who added microencapsulated sea buckthorn extract in muffins, but also with Mihalcea et al. [14], who added microencapsulated sea buckthorn extract in ice cream.

### 3.7. Value-Added Mayonnaise Color Analysis

The sauces were also analyzed in terms of CIELAB colorimetric parameters. The results were expressed as parameters L* (brightness), a* (trend towards red or green), and b* (trend towards yellow or blue). Table 7 shows the sauces’ CIELAB parameter values.

The significant increase of b* value with the powder concentration suggests a tendency to yellow, offered by the biologically active compounds from the microcapsules used as a functional ingredient (*p* < 0.05). Withal, a significant lightness decrease is observed with the powder concentration increase and a tendency to green (Table 7). 

### 3.8. Value-Added Mayonnaise Texture Analysis

In order to estimate the impact of microcapsules over the texture of mayonnaise sauce, the firmness, adhesion, cohesiveness, and chewability were tested using the TPA method. The results of these parameters are presented in Table 8.

The addition of powder determined higher values of the firmness in the mayonnaise sauce compared to the control sample (Table 8). This may be due to the stabilizing compounds from the encapsulation matrix. Blank sample showed a higher porosity than the other variants, which led to significantly lower resistance during compression (*p* < 0.05). Ursache et al. [4] also observed these findings in value-added muffins. In our study, it was also noted that the addition of powder led to improved adhesion. In terms of cohesion and elasticity, no significant differences were observed with the powder addition (*p >* 0.05). 

### 3.9. Value-Added Mayonnaise Sensorial Analysis 

**Figure 3:**
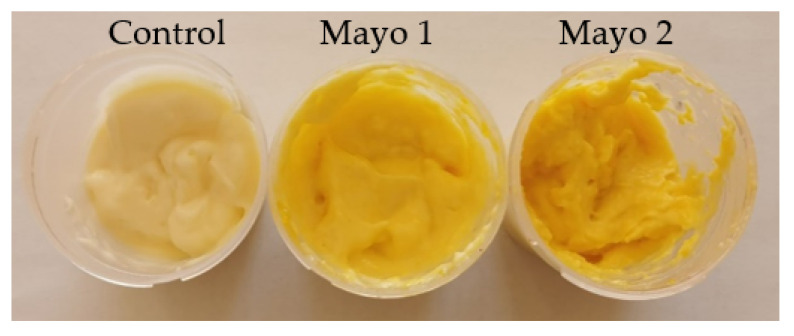
Mayonnaise variants: Control = mayonnaise without the addition of powder; Mayo 1 and Mayo 2 = mayonnaise with 2.5% and 5% powder of sea buckthorn extract.

From a sensory point of view, the sauces were analyzed using a nine attributes scale based on a unit numbering. The attributes pursued were color, aroma, taste, consistency, texture, smell, aftertaste, spreadability, and acceptability (Figure 3). 

**Figure 4:**
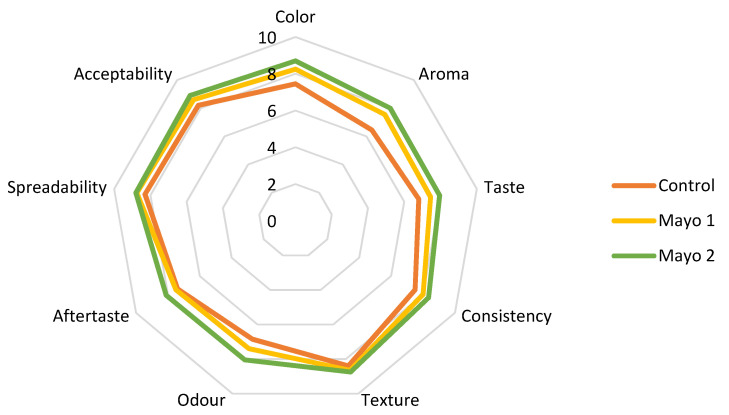
Comparative diagram of the sensory attributes specific to sauces: Control = mayonnaise without the addition of powder; Mayo 1 and Mayo 2 = mayonnaise with 2.5% and 5% powder of sea buckthorn extract.

The average scores obtained from the sensory analysis are shown in Figure 4.

Sample Mayo 2 with 5% sea buckthorn encapsulated extract obtained the highest scores for all nine attributes. However, no significant differences were observed between the scores of Mayo 1 and Mayo 2 (Table 9) (*p* > 0.05). Even if the control sample had the lowest overall scores, in some attributes, such as texture and aftertaste, it was just as appreciated as the other ones. Thus, in terms of texture, odor, aftertaste, and spreadability, the tasters did not find significant differences (*p* > 0.05). 

The panelists reported color differences between the sauces, Mayo 2 being the yellowest one. The mayonnaise sauces with added sea buckthorn powder were evaluated as having a balanced taste, smell, and color. Moreover, the fine, creamy, and fluffy consistency of the sauces was appreciated. All of the proposed samples were positively evaluated by the team of tasters, with no sea buckthorn flavor being perceived.

At the end of the evaluation session, the participants were asked to choose their preferred formulation among the three ones tested. Half of the panelists preferred Mayo 1, while the other half preferred Mayo 2.

## 4. Conclusions

In this study, carotenoids from sea buckthorn extract were successfully encapsulated using a combination of complex coacervation and freeze-drying techniques. Two different combinations of whey proteins and polysaccharides were used as carrier agents. The highest encapsulation efficiency of carotenoids was obtained for P2 (61.17 ± 0.89%), which had a matrix formed by only polysaccharides. Both powders presented high phytochemical content with high antioxidant activity. Slight degradation of phytochemicals was observed during storage, with the increase of powder lightness and decrease of yellowness. In this regard, a 30% decrease in the TC content and 13% in the antioxidant activity of P1 took place, while for P2 13% and 11% decreases in the TC content and antioxidant activity were observed. CLSM revealed the presence of carotenoids anchored within the encapsulation matrices, well individualized but with a tendency to form clusters in both powders. Both matrices increased the bioavailability of carotenoids from sea buckthorn extract in the in vitro digestive system. Thus, P1 presented a maximum release of 82.47 ± 0.22%, while P2 presented a 47.14 ± 0.44% release after 2 h of intestinal digestion. To prove the functionality, different percentages of P2 were evaluated as an ingredient in mayonnaise. The addition of powder improved the carotenoid content and antioxidant activity of the sauces. In addition, the textural analysis suggested that the addition of microencapsulated powder caused the increase of firmness and adhesion. Sensorial analysis showed that panelists appreciated the improved color of the mayonnaise samples. An overall acceptability of the value-added mayonnaise was observed. Therefore, the microencapsulation of sea buckthorn berry extracts and their incorporation in mayonnaise present a promising method for the stabilization of carotenoids in the obtained products. Thus, sea buckthorn extracts may be considered as valuable ingredients for the development of added-value food products.

## Figures and Tables

**Table 1:** Phytochemical characterization of sea buckthorn’s extract and microencapsulated powders.

Sample	Total Carotenoids (mg/g dw)	Antioxidant Activity, (μM TE/g dw)	Encapsulation (μM TE/g dw)	Efficiency, (%)
E	32 ± 1	422 ± 1	-	
P1	2.20 ± 0.13 ^a^	384 ± 2 ^a^	45 ± 2 ^a^	
P2	2.89 ± 0.02 ^b^	454 ± 4 ^b^	61.2 ± 0.9 ^b^	

Values in a column that share a superscript letter (^a, b^) are not significantly different (*p >* 0.05). Measurements are expressed as mean (*n* = 3) ± standard deviation (SD). E = sea buckthorn extract; P1 = powder with 2% WPI and 2% CMC; P2 = powder with 4% alginate, 1% agar, and 4% chitosan.

**Table 2:** Colorimetric parameters of microencapsulated sea buckthorn extract.

Sample	Colorimetric Parameters		
	L*	a*	b*
P1	73.58 ± 0.04 ^a^	8.99 ± 0.02 ^a^	52.4 ± 0.6 ^a^
P2	92.09 ± 0.6 ^b^	4.28 ± 0.05 ^b^	62 ± 1 ^b^

L* = lightness, a* = redness, b* = yellowness; values that share a superscript letter (^a, b^) on a column are not significantly different (*p > 0.05*). Measurements are expressed as mean ± SD of triplicate. P1 = powder with 2% WPI and 2% CMC; P2 = powder with 4% alginate, 1% agar, and 4% chitosan.

**Table 3:** Storage stability of phytochemicals from powders.

Storage Period, Days	P1		P2	
	TC, (mg/g DW)	Antioxidant Activity, (μM Trolox/g dw)	TC, (mg/g DW)	Antioxidant Activity (μM Trolox/g dw)
0	2.20 ± 0.13 ^aA^	384 ± 2 ^aC^	2.89 ± 0.02 ^aB^	453 ± 2 ^aD^
30	2.03 ± 0.10 ^abA^	371.9 ± 0.2 ^aC^	2.74 ± 0.01 ^abB^	451.2 ± 0.3 ^aD^
60	1.79 ± 0.01 ^bcA^	366 ± 8 ^aC^	2.69 ± 0.10 ^abB^	426 ± 7 ^bD^
90	1.54 ± 0.01 ^cA^	335 ± 8 ^bC^	2.52 ± 0.01 ^bB^	404 ± 4 ^cD^

Values from a column that share a superscript lowercase letter (^a, b, c^) are not significantly different (*p* > 0.05). Values from a line that share a superscript capital letter (^A, B, C, D^) are not significantly different (*p* > 0.05). Measurements are expressed as mean ± SD of triplicate. P1 = powder with 2% WPI and 2% CMC; P2 = powder with 4% alginate, 1% agar, and 4% chitosan.

**Table 4:** Storage stability of color from powders.

Storage Time, Days	P1			P2		
	Colorimetric Parameters					
	L*	a*	b*	L*	a*	b*
0	73.58 ± 0.04 ^a^	8.99 ± 0.02 ^a^	52.4 ± 0.6 ^a^	92.1 ± 0.6 ^a^	4.28 ± 0.05 ^a^	62 ± 1 ^a^
30	78.02 ± 0.14 ^a^	9.4 ± 0.4 ^a^	50.4 ± 0.5 ^ab^	93 ± 1 ^b^	5.3 ± 0.3 ^a^	60.7 ± 0.4 ^ab^
60	90.53 ± 0.05 ^b^	9.4 ± 0.4 ^a^	48.6 ± 0.6 ^b^	92.4 ± 0.6 ^b^	6.43 ± 0.19 ^b^	59.4 ± 0.3 ^ab^
90	91.3 ± 0.5 ^c^	10.9 ± 0.9 ^a^	45.6 ± 0.5 ^c^	99 ± 1 ^b^	6.94 ± 0.04 ^c^	58.3 ± 0.5 ^b^

L* = lightness, a* = redness, b* = yellowness; P1 = powder with 2% WPI and 2% CMC; P2 = powder with 4% alginate, 1% agar, and 4% chitosan. Values from a column that share a superscript letter (^a, b, c^) are not significantly different (*p* > 0.05). Measurements are expressed as mean ± SD of triplicate.

**Table 5:** Physico-chemical characteristics of value-added mayonnaise.

Physico-Chemical Characteristics	Control	Mayo 1 (2.5%)	Mayo 2 (5%)
Proteins, g/100 g	8.06 ± 0.07 ^a^	6.94 ± 0.02 ^b^	6.93 ± 0.04 ^b^
Lipids, g/100 g	72.0 ± 0.2 ^ab^	72.7 ± 0.3 ^a^	71.3 ± 0.2 ^b^
Carbohydrates, g/100 g	2.5 ± 0.2 ^a^	2.1 ± 0.2 ^a^	2.61 ± 0.05 ^a^
Humidity, g/100 g	15.58 ± 0.04 ^a^	16.28 ± 0.10 ^b^	16.98 ± 0.12 ^c^
Ash, g/100 g	1.84 ± 0.02 ^a^	1.96 ± 0.04 ^a^	2.2 ± 0.2 ^a^
Energetic value, %kcal	713.2 ± 0.7 ^a^	713 ± 2 ^a^	702± 3 ^b^
kJ	2984 ± 3 ^a^	2985 ± 10 ^a^	2938 ± 11 ^b^

Values from a line that share a superscript letter (^a, b, c^) are not significantly different (*p* > 0.05). Measurements are expressed as mean ± SD of triplicate. Mayo 1 = mayonnaise sauce with 2.5% encapsulated sea buckthorn extract; Mayo 2 = mayonnaise sauce with 5% encapsulated sea buckthorn extract.

**Table 6:** Phytochemical characteristics of value-added mayonnaise.

Phytochemical Characteristics	Control	Mayo 1 (2.5%)	Mayo 2 (5%)
TC, mg /100 g dw	0.26 ± 0.07 ^a^	1.23 ± 0.15 ^b^	1.85 ± 0.04 ^c^
Antioxidant activity, μM Trolox/g dw	10.0 ± 0.6 ^a^	152.1 ± 0.4 ^b^	293 ± 3 ^c^

Values from a line that share a superscript letter (^a, b, c^) are not significantly different (*p* > 0.05). Measurements are expressed as mean ± SD of triplicate. Mayo 1 = mayonnaise sauce with 2.5% encapsulated sea buckthorn extract; Mayo 2 = mayonnaise sauce with 5% encapsulated sea buckthorn extract.

**Table 7:** Color characteristics of value-added mayonnaise.

Sample	L*	a*	b*
Control	65.7 ± 0.6 ^a^	−1.01 ± 0.13 ^a^	30 ± 1 ^a^
Mayo 1 (2.5%)	61 ± 1 ^b^	−0.53 ± 0.02 ^b^	42.9 ± 0.8 ^b^
Mayo 2 (5%)	54 ± 1 ^c^	−0.34 ± 0.02 ^b^	57 ± 2 ^c^

L* = lightness, a* = redness, b* = yellowness; Mayo 1 = mayonnaise sauce with 2.5% encapsulated sea buckthorn extract; Mayo 2 = mayonnaise sauce with 5% encapsulated sea buckthorn extract. Values from a column that share a superscript letter (^a, b, c^) are not significantly different (*p* > 0.05). Measurements are expressed as mean ± SD of triplicate.

**Table 8:** Textural characteristics of value-added mayonnaise.

Textural Parameters	Control	Mayo 1 (2.5%)	Mayo 2 (5%)
Firmness, N	0.22 ± 0.01 ^a^	0.23 ± 0.03 ^a^	0.34 ± 0.03 ^b^
Adhesion, mJ	1.63 ± 0.15 ^a^	2.32 ± 0.12 ^b^	3.4 ± 0.4 ^c^
Cohesion	0.68 ± 0.02 ^a^	0.65 ± 0.03 ^a^	0.62 ± 0.05 ^a^
Elasticity, mm	11.1 ± 0.2 ^a^	10.7 ± 0.7 ^a^	11.0 ± 0.3 ^a^

Values from a line that share a superscript letter (^a, b, c^) are not significantly different (*p* > 0.05). Measurements are expressed as mean ± SD of duplicate. Mayo 1 = mayonnaise sauce with 2.5% encapsulated sea buckthorn extract; Mayo 2 = mayonnaise sauce with 5% encapsulated sea buckthorn extract.

**Table 9:** Sensory attributes values given by the panelists to sauces: Control = mayonnaise without the addition of powder; Mayo 1 and Mayo 2 = mayonnaise with 2.5 and 5% powder of sea buckthorn extract.

Sensory Attributes	Control	Mayo 1 (2.5%)	Mayo 2 (5%)
Color	7 ± 1 ^a^	8.3 ± 0.7 ^b^	8.7 ± 0.5 ^b^
Aroma	6 ± 1 ^a^	7.6 ± 0.8 ^b^	8.0 ± 0.9 ^b^
Taste	6.8 ± 0.9 ^a^	7.5 ± 0.9 ^b^	8.0± 0.5 ^b^
Consistency	7.5 ± 0.9 ^a^	8.0 ± 0.7 ^ab^	8.4 ± 0.6 ^b^
Texture	8.4 ± 0.8 ^a^	8.7 ± 0.6 ^a^	8.8 ± 0.4 ^a^
Odor	6.9 ± 1.3 ^a^	7.40 ± 0.18 ^ab^	8.1 ± 0.6 ^b^
Aftertaste	7.4 ± 0.8 ^a^	7.50 ± 0.19 ^a^	8 ± 1 ^a^
Spreadability	8 ± 1 ^a^	8.8 ± 0.4 ^a^	8.8 ± 0.4 ^a^
Acceptability	8.2 ± 0.6 ^a^	8.6 ± 0.5 ^b^	8.9 ± 0.3 ^b^

Values from a line that share a superscript letter (^a, b^) are not significantly different (*p* > 0.05). Measurements are expressed as mean ± SD of duplicate. Mayo 1 = mayonnaise sauce with 2.5% encapsulated sea buckthorn extract; Mayo 2 = mayonnaise sauce with 5% encapsulated sea buckthorn extract.

## Data Availability

Not applicable.
